# Single-step synthesis of a new series of *meso di*-Mannich bases from the cyclic aminal (2*S*,7*R*,11*S*,16*R*)-1,8,10,17-tetraazapentacyclo[8.8.1.1.^8,17^0.^2,7^0^11,16^]icosane and *p*-substituted phenols

**DOI:** 10.1186/1752-153X-7-100

**Published:** 2013-06-11

**Authors:** Augusto Rivera, Diego Quiroga, Jaime Ríos-Motta, Václav Eigner, Michal Dušek

**Affiliations:** 1 Departamento de Química, Facultad de Ciencias, Universidad Nacional de Colombia, Ciudad Universitaria, Carrera 30 N° 45-03, Bogotá, D.C., Colombia; 2 Department of Solid State Chemistry, Institute of Chemical Technology, Technická 5, Prague 166 28, Czech Republic; 3 Institute of Physics AS CR, v.v.i., Na Slovance 2, Prague 8 182 21, Czech Republic

## Abstract

**Background:**

The results presented herein show that the cyclic aminal (2*S*,7*R*,11*S*,16*R*)-1,8,10,17-tetraazapentacyclo[8.8.1.1.^8,17^0.^2,7^0^11,16^]icosane (6), derived from *cis*-(*meso*)-1,2-diaminocyclohexane and formaldehyde, is a suitable substrate for the preption of a series of *cis-meso* Mannich bases such as 8a-l by reaction with *p*-substituted phenols 7a-l in basic media. These compounds are valuable synthetic products and may find application in asymmetric catalysis.

**Results:**

The products were characterized principally by NMR and IR spectroscopy. Both the benzylic and aminalic protons of the perhydrobenzimidazolidine moiety were diastereotopic due to the presence of stereogenic nitrogen centers. The occurrence of intramolecular hydrogen bonding interactions was confirmed by the broad OH stretching vibration band in the IR spectra. Vibrational spectra were calculated using B3LYP at 6-31G(d,p) level, and the calculated frequencies for the νOH vibrations were compared to those of the experimental spectra. Hydrogen bonding interactions in the solid state were observed through the X-ray crystallography of 8j. Additionally, Mulliken charges and Fukui indices for 6 were calculated as theoretical descriptors of electrophilicity.

**Conclusion:**

A new series of *meso* Mannich bases called 4,4′-disubstituted-2,2′-{[(3a*R*,7a*S*)-2,3,3a,4,5,6,7,7a-octahydro-1*H*-1,3-benzimidazole-1,3-diyl]*bis*(methylene)} diphenols (8a-l) which are derived from *cis*-(*meso*)-1,2-diaminocyclohexane, were obtained from cyclic aminal 6. These results confirmed the behavior of 6 as an electrophilic preformed reagent in Mannich reactions in basic media.

## 

Mannich bases are an interesting family of compounds in organic chemistry, and these compounds have been widely used in diverse chemistry fields due to their biological and pharmacological activities [1-4]. Moreover, the Mannich bases have been used as molecular models for studies of intramolecular proton transference processes [5,6] due to their interesting thermodynamic stability. Our interest in Mannich bases is focused on their application as model systems for studying inter and intramolecular hydrogen bond interactions between the phenolic OH atoms and the amine N atoms in phenolic derivatives [7-9]. Mannich bases can be obtained by multi-component Mannich condensation reactions between an amine, formaldehyde and *active hydrogen compounds* in acidic media in low to moderate yields [10]. The main subject of research is the reactivity of cyclic aminals toward nucleophiles and electrophiles. Our results using phenols as nucleophiles have led to the synthesis of *di*-Mannich bases, demonstrating that cyclic aminals behave as preformed electrophilic reagents.

One example of the application of cyclic aminals as Mannich base precursors is the chemical reactivity of 1,3,6,8-tetraazatricyclo[4.4.1.1^3,8^]dodecane (TATD, 1), which by reaction with *p*-substituted phenols in basic media, leads to the synthesis of 2,2′-(imidazolidine-1,3-diyl*bis*(methylene))*bis*(4-substitutedphenols) (2) (Figure 1), an interesting family of *di*-Mannich bases that show a stability due to the presence of two OH---N intramolecular hydrogen bonds [11-15].

**Figure 1:**
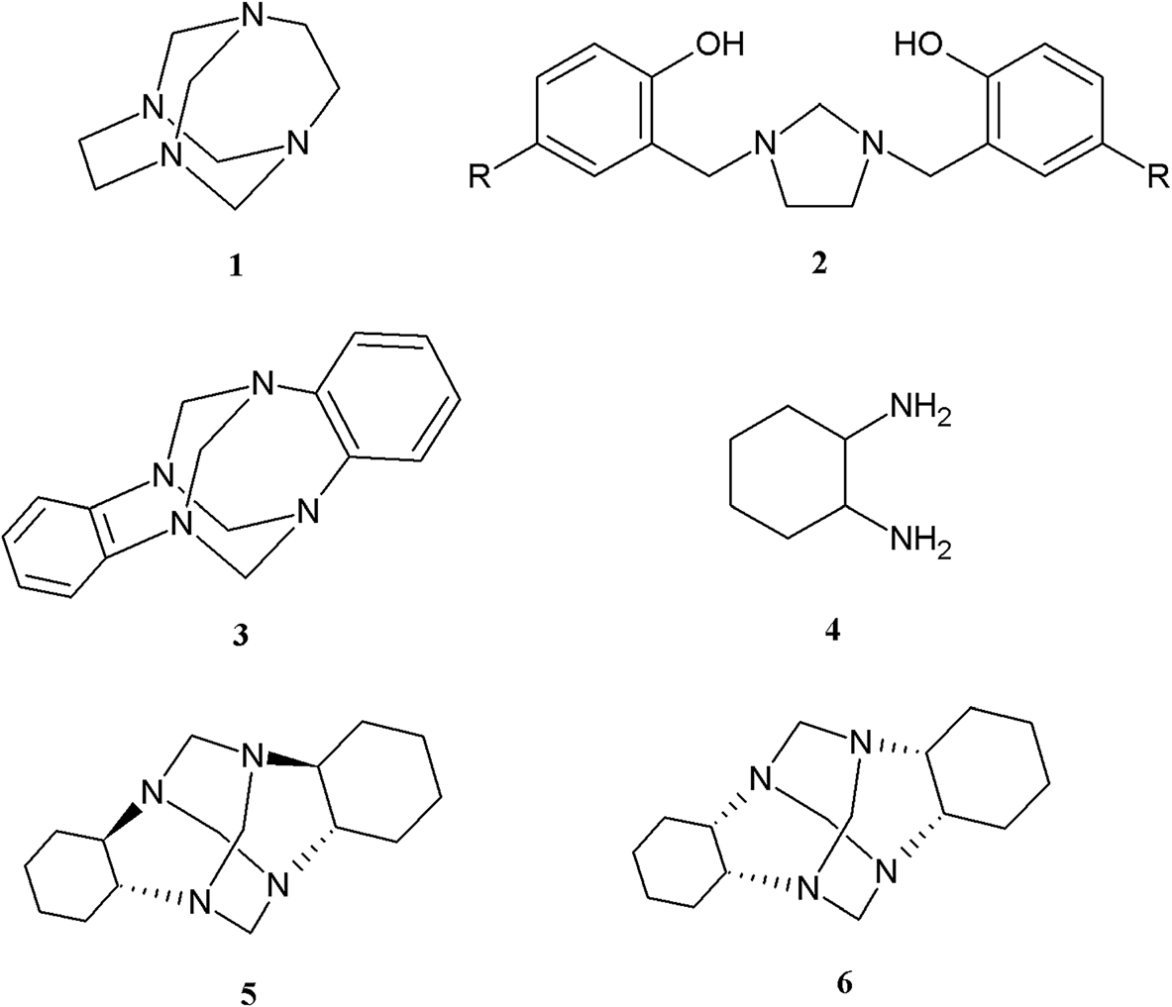
Structures of 1-6.

We believe that the electrophilic behavior of cyclic aminals is determined by their structural features, especially the presence of 1,1- and 1,2-diamine functionalities. The interactions of the nonbonding electron pairs of nitrogen atoms and the presence or absence of stereoelectronic effects play an important role in the relative stability and chemical reactivity of this type of cyclic aminal [16]. Our hypothesis was supported by experiments using a cyclic aminal 6*H*,13*H*-5:12,7:14-dimethanedibenzo[*d*,*i*][1,3,6,8]tetraazecine DMDBTA (3) (Figure 1), a cyclic aminal analog similar to TATD 1. All the efforts to synthesize type (2) *di*-Mannich bases were unsuccessful, and *N*-substituted benzimidazoles were obtained using electron-rich phenols [17], due to spontaneous reaction under air oxidative conditions and thermodynamically driving by aromatisation. To obtain cyclic aminals with molecular structures that do not involve electron delocalization of the nonbonding pairs, we employed aliphatic 1,2-diamines such as 1,2-diaminocyclohexane (4).

1,2-Diaminocyclohexane 4 is an organic compound with a 1,2-disubstituted cyclohexane type structure and two primary amino groups attached to two stereogenic centers (Figure 1). For this compound exists the (*R*, *R*)-, (*S*, *S*)-, and (*S*, *R*) stereoisomers. Both (*R*, *R*)- and (*S*, *S*)-4 have *trans* isomerism while (*S*, *R*)-4 has *cis* isomerism. *Trans*-(*R*, *R*)- and *trans*-(*S*, *S*)-4, which presents *C*_*2*_ symmetry, have been widely used in asymmetric synthesis because their two primary amino groups possess non-bonding orbitals allowing their use as a ligand for transition metals [18-20]. Although *cis*-1,2-diaminocyclohexane stereoisomer with average *C*_*2v*_ symmetry is less stable than *trans* stereoisomers, *cis* stereoisomer has also been studied for the preption of metal complexes that can be employed in a variety of applications, including the synthesis and pharmacological activity of inorganic complexes of platinum (II), cobalt (II), nickel (II) and copper (II) [21-25]. Both *trans* and *cis* isomers have been used to obtain cyclic aminals (2*R*,*7R*,11*S*,16*S*)-1,8,10,17-tetrazapentacyclo [8.8.1.1^8,17^.0^2,7^.0^11,16^]icosane (5) and (2*S*,*7R*,11*S*,16*R*)-1,8,10,17-tetrazapentacyclo[8.8.1.1^8,17^.0^2,7^.0^11,16^]icosane (6) (Figure 1), respectively [26,27].

Moreover, X-ray analysis suggested that compound 6 could be a reactive precursor, considering the presence of strained cyclohexane rings; the geometry of which is nearly planar and presented internal bond angles between 113.9(7)° and 124.6(8)° [27]. The bond angles confirmed that the aminal groups displayed a distorted tetrahedral geometry of 119.9(9)° to 120.4(7)° due to the *cis* configuration in the diamine moiety [27]. To obtain new Mannich bases involving fused rings in central chiral-based structures and to understand the stereoelectronic effect of nonbonding pairs in cyclic aminals, we studied Mannich type reactions with cyclic aminals derived from chiral diamines. We opened the study with new cyclic aminal 6 and various nucleophiles, beginning with the reaction between 6 and *p*-substituted phenols 7a-l to afford 4,4′-disubstituted-2,2′-{[(3a*R*,7a*S*)-2,3,3a,4,5,6,7,7a-octahydro-1*H*-1,3-benzimidazole-1,3-diyl]*bis*(methylene)}di phenols (8a-l), a new family of Mannich bases (Scheme 1). In this article, we discuss the synthetic methodology and characterization of these compounds using FT-IR and NMR analysis, as well as X-ray diffraction.

**Scheme 1:**
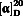
**Reaction between cyclic aminal 6 and *p*-substituted phenols 7a-l.**

### Results and discussion

Cyclic aminal 6 was prepared in high yield (90%) by the condensation of *cis*-(*meso*)-1,2-diaminocyclohexane with pformaldehyde in *N*,*N*-DMF, according to the previously reported procedure [27].

The overall procedure for the synthesis of Mannich bases (8a-l) is depicted in Scheme 1. The isolated products were characterized by FT-IR and uni- and bi-dimensional NMR experiments. All of the compounds were identified as 4,4′-disubstituted-2,2′-{[(3a*R*,7a*S*)-2,3,3a,4,5,6,7,7a-octahydro-1*H*-1,3-benzimidazole-1,3-diyl]*bis*(methylene)}diphenols (8a-l). Reactions between 6 and *p*-substituted phenols 7a-l were carried out at 40°C in 1,4-dioxane: water at a volumetric ratio of 3:2 Table 1. Apparently, water is necessary for such reactions because attempts to obtain compounds 8a-l under aprotic conditions were unsuccessful. The reactions showed *ortho*-regioselective aminomethylation of the aromatic ring of the phenols. The formation of compounds 8a-l can be easily explained by analogy with the mechanism proposed for the reaction of cyclic aminal 1 with phenols [11,12].

**Table 1:** Substrate scope of Mannich bases synthesis

**Entry**	**Compound**	**R**	**Product**	**m.p. (°C)**	${\left[\alpha \right]}_{D}^{20}$ **c = 0.6, CH**_**2**_**Cl**_**2**_	**Yield (%)**
**1**	**7a**	F	**8a**	168-169	+5.2	20
**2**	**7b**	Cl	**8b**	189-190	+5.7	34
**3**	**7c**	Br	**8c**	183-184	+6.0	41
**4**	**7d**	I	**8d**	175-177	+7.1	20
**5**	**7e**	COOMe	**8e**	128-130	+6.3	17
**6**	**7f**	COOEt	**8f**	151-153	+6.8	19
**7**	**7 g**	COOPr	**8 g**	118-119	+5.5	18
**8**	**7 h**	COOBu	**8 h**	127-129	+4.9	19
**9**	**7i**	H	**8i**	146-148	+6.8	20
**10**	**7j**	Me	**8j**	162-163	+7.2	46
**11**	**7 k**	t-Bu	**8 k**	141-143	+6.3	49
**12**	**7 l**	OMe	**8 l**	132-134	+5.9	45

Table 1 shows melting points and yield for each new compound, indicating the *p*-substituent in aromatic rings in both phenols and compounds 8a-l.

The experimental FT-IR spectra of the Mannich bases (8a-l) (Table 2) showed a broad absorption band between 3300–2350 cm^-1^, which was assigned to the O–H stretching vibration of the phenolic moiety and is a result of OH•••N hydrogen bonding interactions, suggesting that the proton remains covalently bonded to the hydroxyl group and that proton transfer to the amino group did not occur. To understand the effect of hydrogen bonding interactions on the molecular structure of these compounds, we performed theoretical calculations. Thus, geometry optimizations and vibrational frequencies of the products were performed in Gaussian 1998 using DFT B3LYP methods at the 6-31G(d,p) level [28].

**Table 2:** Assignment of vibrational modes of compounds 8a-l

**Product**	**1**	**2**	**3**	**4**	**5**	**6**	**7**	**8**	**9**
**8a**	3050	2848	1630	1448	1387	1194	1289	1063	772
			1495			1124			737
**8b**	3053	2922	1605	1444	1385	1170	1272	1069	764
		2850	1480			1069			680
**8c**	3054	2933	1636	1444	1384	1169	1283	1071	761
		2849	1476			1112			673
**8d**	3070	2931	1603	1440	1367	1179	1262	1039	764
		2854	1476			1129			
**8e**	3042	2928	1609	1449	1384	1190	1293	1097	791
		2854	1497			1110			771
**8f**	3040	2980	1612	1452	1384	1178	1285	1098	840
		2931	1495			1124		1058	770
		2857							
**8 g**	3049	2932	1613	1448	1384	1178	1270	1037	840
		2875	1497			1127			771
**8 h**	3056	2961	1613	1449	1383	1177	1258	1059	874
		2931	1497			1127			770
		2858							
**8i**	3037	2946	1618	1469	1393	1096	1243	1070	742
		2913	1486						
		2874							
**8j**	3060	2962	1613	1449	1394	1117	1259	1067	817
		2934							773
		2862							
**8 k**	3060	2962	1613	1449	1394	1122	1252	1068	857
		2934	1502						824
		2862							
**8 l**	3051	2962	1617	1465	1384	1068	1289	1039	820
		2923	1497						775
		2850							

Vibrational modes: 1) Stretching frequency of O-H bonds, 2) Stretching frequency of the C_*sp3*_-H bonds, 3) symmetric and asymmetric stretching frequency of the C-C bond, 4) Flexion frequency of the C-H bond in aromatic rings, 5) out of plane flexion frequency of methylene groups, 6) Stretching frequency of the C-N bonds, 7) Stretching frequency of the C-O bonds, 8) in the plane deformation of the C-H bonds in aromatic rings, 9) out of plane deformation of the C-H bonds in aromatic rings.

Table 2 shows the measured vibrational modes of compounds 8a-l, obtained using FT-IR spectroscopy.

The computational calculations showed that the calculated frequencies of the νOH vibrations of compounds 8a-l (3335 and 3345 cm^-1^) were higher than that of the experimental spectra, where these vibrations appeared as very broad and weak absorptions. As described by several authors who studied similar compounds [29,30], these differences can be attributed to the strong anharmonicity of this type of vibration, which was not included in the calculation process. The calculated frequencies of the aromatic and fused rings of the perhydrobenzimidazolidine moiety were located in the expected ranges. For the calculated C-O stretching frequencies of the phenol precursors (7a-l) we noted that the calculated values are systematically lower than the experimental results of respective product (8a-l), suggesting that the C-O bond length was shortened due to intramolecular hydrogen bonding (Table 2).

However, the use of this band to understand the effects of hydrogen bonding in the structures of 8a-l is limited due to its low intensity and the presence of aromatic ring deformation vibrations in this region, which prevented assignment. The ^13^C NMR spectra of all of the synthesized compounds 8a-l (Table 3) showed two signals between 21.0 and 25.0 ppm, which were assigned to the methylene carbon atoms of the cyclohexane ring. The signal at 61.0 ppm was assigned to the methine chiral carbon atoms. Using HMQC and HMBC bidimensional experiments, the signal at 73.5 ppm was assigned to the aminalic carbon atom (N–CH_2_–N). Moreover, the benzylic carbon atoms showed a signal at 55.0 ppm. The carbon atoms of the aromatic rings appeared as six signals between 115 and 158 ppm. The ^1^H NMR spectra of compounds 8a-l (Table 4) showed that the hydrogen atoms in the ArCH_2_ group were diastereotopic, presenting two doublets around 3.64 and 4.04 ppm and a geminal coupling ^2^*J*_H,H_ constant of 14.0 Hz. The ^1^H NMR signals above 6.0 ppm allowed us to determine the substitution pattern of the aromatic rings and confirmed the *ortho*-regioselective aminomethylation of the Mannich bases.

**Table 3:** **Assignment of the**^**13**^**C NMR spectra of the compounds 8a-l**

**Product**	**R**	**C1, C1′**	**C2, C2′**	**C3, C3′**	**C4, C4′**	**C5, C5′**	**C6, C6′**	**C2**	**ArCH**_**2**_**N**	**C3a, C7a**	**C4, C5 C6, C7**
**8a**	F	153.4 *d*	122.0 *d*	114.7 *d*	156.0 *d*	115.4 *d*	117.0 *d*	73.4	55.0	61.1	21.5
											24.7
**8b**	Cl	156.2	122.5	128.0	123.9	129.0	117.6	73.4	55.0	61.1	21.5
											24.7
**8c**	Br	156.9	123.2	131.0	111.2	132.1	118.3	73.6	55.0	61.2	21.6
											24.8
**8f**	(C = O)OEt (14.5, 60.8, 166.5)	162.1	120.9	130.3	121.8	131.4	116.3	73.7	55.4	61.2	21.6
											24.9
**8 g**	(C = O)OPr (10.7, 22.3, 66.4, 166.5)	162.0	121.8	130.5	121.8	131.5	116.5	73.4	56.3	61.2	21.6
											24.9
**8 h**	(C = O)OBu (13.9, 19.4, 31.0, 64.6, 166.6)	162.2	121.0	130.2	121.8	131.4	116.3	73.7	55.5	61.2	21.7
											24.9
**8i**	H	157.6	121.3	128.2	119.3	129.1	116.1	73.4	55.4	61.0	21.6
											24.6
**8j**	Me (20,4)	155.2	121.0	128.8	128.4	129.5	115.9	73.4	55.3	61.0	20.4
											21.7
**8 k**	*t*-Bu (31.6, 33.9)	155.2	120.4	125.0	142.0	125.8	115.5	73.5	55.9	61.0	21.7
											24.6
**8 l**	OMe (55.7)	152.6	121.9	114.1	151.3	114.1	116.6	73.4	55.4	61.1	21.6
											24.6

Table 3 represents all the measured signals for carbon atoms in compounds 8a-l in the ^13^C NMR spectra and their assignation in the molecular structure.

**Table 4:** **Assignment of the**^**1**^**H NMR spectra of compounds 8a-l**

**Product**	**R**	**Ar-OH**	**H-5.**	**H-3,**	**H-6,**	**H-2,**	**ArCH**_**2**_**N**	**H-3a,**	**H-4, H-5**
			**H-5′**	**H-3′**	**H-6′**	**H-2′**		**H7a**	**H-6, H-7**
**8a**	F	10.34, *bs*	6.87, *td, J* = 8.0 Hz, *J* = 8.2 Hz, *J* = 3.1 Hz	6.70, *dd, J* = 8.0 Hz, *J* = 2.8 Hz	6.76, *dd, J* = 8.0 Hz, *J* = 4.8 Hz	3.39,*d,* 3.84, *d, J =* 6.4 Hz	3.63, *d,* 4.03, *d, J =* 14.0 Hz	3.11, *t, J* = 4.0 Hz	1.30-1.80, *m*
**8b**	Cl	10.63, *bs*	7.15, *dd, J* = 8.6 Hz, *J* = 2.6 Hz	6.97, *d, J* = 2.5 Hz	6.78, *d, J* = 8.6 Hz	3.39, *d,* 3.85, *d J* = 6.6 Hz	3.64, *d,* 4.04, *d J* = 14.0 Hz	3.11, *t, J* = 4.1 Hz	1.30-1.80, *m*
**8c**	Br	10.55, *bs*	7.26, *dd, J* = 8.6 Hz, *J* = 2.4 Hz	7.09, *d, J* = 2.4 Hz	6.72, *d, J* = 8.6 Hz	3.36, *d,* 3.84, *d J* = 6.6 Hz	3.62, *d,* 4.02, *d, J* = 13.9 Hz	3.09, *t, J* = 4.0 Hz	1.30-1.80, *m*
**8d**	I	---	7.45, *d, J* = 8.8 Hz	7.27, *s*	6.62, *d, J* = 8.5 Hz	3.62, *d,* 3.85, *d, J* = 8.0 Hz	3.62, *d,* 4.01, *d, J* = 13.9 Hz	3.11, *t, J* = 4.0 Hz	1.30-1.80, *m*
**8e**	(C = O)OMe (3.87, *s*)	10.79, *bs*	7.96, *dd, J* = 8.4 Hz, *J* = 2.1 Hz	7.74, *d, J* = 2.1 Hz	6.87, *d, J* = 8.8 Hz	3.42, *d,* 3.85, *d, J* = 8.0 Hz	3.67, *d,* 4.06, *d, J* = 14.0 Hz	3.11, *t, J* = 4.0 Hz	1.30-1.80, *m*
**8f**	(C = O)OEt (1,35, *t*; 4,31, *q*)	----	7.88, *dd, J* = 8.5 Hz, *J* = 2.1 Hz	7.70, *d, J* = 2.0 Hz	6.83, *d, J* = 8.5 Hz	3.37, *d,* 3.86, *d, J* = 6.6 Hz	3.72, *d,* 4.09, *d, J* = 13.9 Hz	3.11, *t, J* = 4.0 Hz	1.30-1.80, *m*
**8 g**	(C = O)OPr (1.01, *t*; 1.75, *m*; 4.22, *t*)	----	7.89, *dd, J* = 8.5 Hz *J* = 2.1 Hz	7.71, *d, J* = 1.9 Hz	6.86, *d, J* = 8.5 Hz	3.44, *d,* 3.91, *d, J* = 6.2 Hz	3.75, *d,* 4.10, *d, J* = 13.8 Hz	3.15, *t, J* = 4.0 Hz	1.30-1.80, *m*
**8 h**	(C = O)OBu (0.96, *m*; 1.44, *m*; 1.71, *qn*; 4.26, *t*)	----	7.88, *dd, J* = 8.5 Hz, *J* = 2.1 Hz	7.69, *d, J* = 2.1 Hz	6.83, *d, J* = 8.5 Hz	3.36, *d,* 3.85, *d, J* = 6.6 Hz	3.72, *d,* 4.09, *d, J* = 13.9 Hz	3.10, *t, J* = 4.0 Hz	1.30-1.80, *m*
**8i**	H (6.77, *td*)	10.60, *bs*	7.17, *dd, J* = 8.0 Hz *J* = 1.2 Hz	6.96, *d, J* = 7.2 Hz	6.82, *dd, J* = 8.1 Hz, *J* = 1.0 Hz	3.42, *d* 3.84, *d, J* = 6.6 Hz	3.67, *d,* 4,06, *d, J* = 13.8 Hz	3.11, *t, J* = 4.0 Hz	1.30-1.80, *m*
**8j**	Me (2.24, *s*)	10.62, *bs*	6.99, *d, J* = 8.2 Hz	6.79, *s*	6.74, *d, J* = 8.2 Hz	3.44, *d* 3.83, *d, J* = 6.5 Hz	3.64, *d,* 4.04, *d, J* = 13.7 Hz	3.12, *t, J* = 4.1 Hz	1.30-1.80, *m*
**8 k**	*t*-Bu (1.27, *s*)	10.62, *bs*	7.19, *dd, J* = 8.5 Hz, *J* = 2.4 Hz	6.96, *d, J* = 2.4 Hz	6.75, *d, J* = 8.5 Hz	3.47, *d* 3.86, *d, J* = 6.6 Hz	3.67, *d* 4.07, *d, J* = 13.8 Hz	3,11, *t, J* = 4.3 Hz	1.30-1.80, *m*
**8 l**	OMe (3.71, *s*)	10.15, *bs*	6.73, *d, J* = 8.8 Hz	6.53, *d, J* = 2.0 Hz	6.75, *d, J* = 8.8 Hz	3.42, *d* 3.83, *d, J* = 6.5 Hz	3.62, *d* 4.02, *d, J* = 13.7 Hz	3,11, *t, J* = 4.1 Hz	1.30-1.80, *m*

Table 4 shows all the measured signals for hydrogen atoms in compounds 8a-l in the ^1^H NMR spectra and their assignation in the molecular structure.

In the ^1^H NMR spectra of the products obtained from *p*-substituted phenols, signals as singlets and doublets with *meta* coupling (around 2.0 Hz) in an ABX system were observed and were assigned to hydrogen atoms in the *ortho* position with respect to the methylene group and the *meta* position with respect to the hydroxyl group. In addition, signals as doublets and doublets of doublets with typical *ortho* and *meta* coupling constants (8.4 Hz and 2.4 Hz, respectively), were also detected. The ^1^H NMR spectrum of compound (8i) showed an ABCD coupling system with a triplet of doublets around 6.77 ppm with a *meta* coupling constant of ^4^ *J* = 1.1 Hz and an *ortho* coupling constant of ^3^ *J* = 7.4 Hz with the signal at 6.96 ppm, which appeared as a doublet and was assigned to the R = H atoms and the hydrogen atom in the *ortho* position with respect to the methylene group attached to the aromatic ring. However, the signal at 6.82 ppm appeared as a doublet of doublets with a *meta* coupling constant ^4^ *J* = 1.0 Hz and an *ortho* coupling constant of ^3^ *J* = 8.1 Hz with the signal at 7.17 ppm, which appeared as a multiplet and was assigned to the hydrogen in the *ortho* position and the hydrogen atoms in the *meta* position with respect to the hydroxyl group, respectively. The hydrogen atoms of the hydroxyl groups were shifted to a low field (above 10.6 ppm), confirming the existence of intramolecular hydrogen bonding interactions.

The cyclohexane ring can be identified in the ^1^H NMR spectra as four multiplet signals between 1.39 and 3.11 ppm. These hydrogen atoms are diastereotopic due to the presence of chiral carbon atoms and stereogenic nitrogen centers. For the signal at 3.11 ppm, which presented a vicinal ^1^H/^1^H coupling constant of 4.0 Hz, we calculated the torsional angles between the methine hydrogens and methylene hydrogens using MestReJ software, which employs the modified Karpluss relation and the dependence of the coupling constant on the torsion angle [31]. The averages over all of the structures (8a-l) were 120° (*α-eq*,*β-eq*) and 52° (*α-eq*,*α-ax*), respectively. The signals that appeared as doublets at 3.39 and 3.85 ppm with a coupling constant of 6.5 Hz were assigned as the aminalic hydrogen atoms. This experimental evidence is in good agreement with the proposed molecular structures of compounds 8a-l, which belong to the *C*_*1*_ symmetric chiral point group and presents the lowest degree of symmetry. Because the aminalic protons have a distinct chemical environment due to their spatial orientation (*axial* and *equatorial* dispositions in the imidazolidine ring, respectively), the deprotection of the *equatorial* proton, which was shifted to higher frequencies is evidenced in the doublet signals septed by 0.42 ppm. These results can be explained considering a hyperconjugation effect, which can be attributed to the interaction between nitrogen lone pairs and antibonding orbital σ*_C,Hax_ (n_N_ → σ*_C,Hax_), the latter of which was *synperiplanar* to the nitrogen lone pairs (Figure 2) [32]. A consequence of this effect is the elongation of the C-H_ax_ bond with respect to the C-H_eq_ bond, which was equal to 0.02 Å, as observed in the optimized molecular structure.

**Figure 2:**
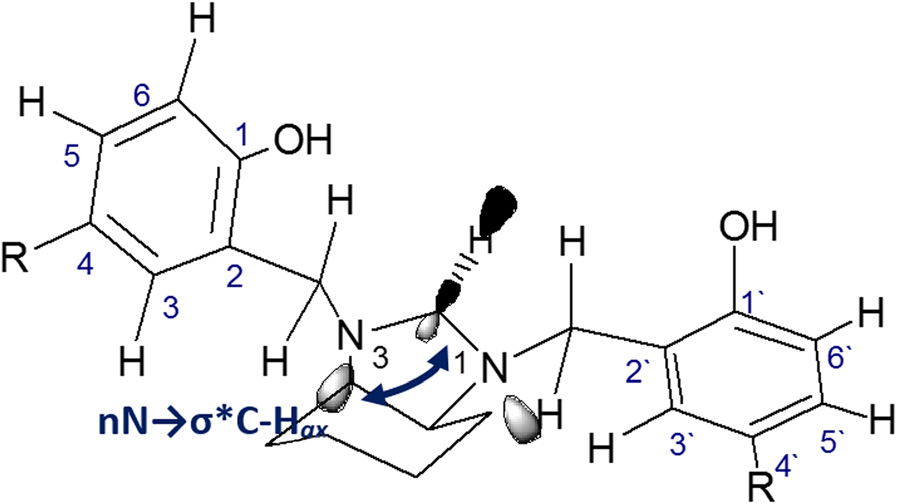
Hyperconjugation in compounds 8a-l.

To understand the incidence of *cis* isomerism in the molecular structure of compounds 8a-l, efforts were made to obtain monocrystals suitable for X-ray diffraction analysis. A monocrystal of compound 8j was obtained via recrystallization from a mixture of chloroform and methanol. Compound 8j exists mainly with the OH groups engaged in an intramolecular hydrogen bond with the N atoms of imidazolidine ring. The molecular structure of compound 8j (Figure 3) is stabilized by two O—H · · · N intramolecular hydrogen bonds.

**Figure 3:**
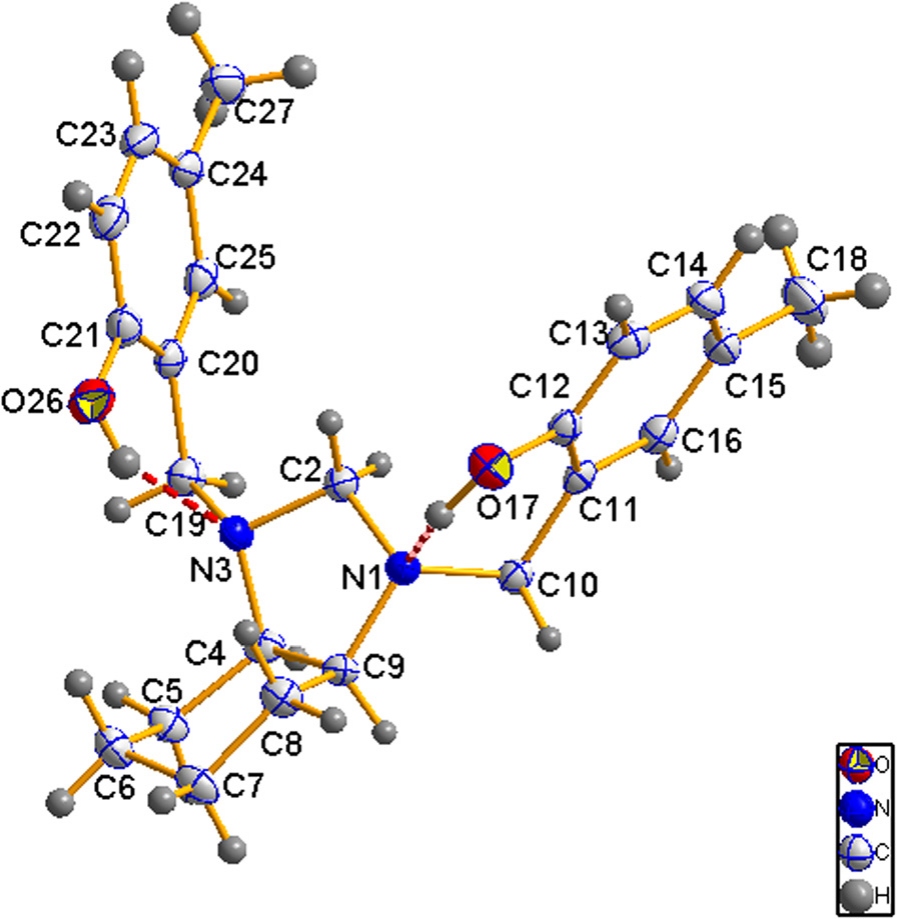
Molecular structure of 8j (ellipsoids are drawn with 50% probability).

The imidazolidine ring adopts an envelope conformation, and the fused six-membered ring adopts a chair conformation. The dihedral angle between the mean planes of these rings, defined by C9-N1-C2 and C5-C4-C8, is 47.84(12)°. The substituents on the N atoms of the five-membered ring are arranged *syn* with respect to the central ring. The phenyl rings are oriented at angles of 82.15 (14)° (C11-C16) and 83.97 (16)° (C20-C25) with respect to the mean plane of the heterocyclic ring, defined by N1—C2—C9. The two phenyl rings form a dihedral angle of 41.25 (9)°.

To understand the relationship between the molecular structure and observed reactivity of 6, we used two approaches, including: (a) the correlation between the electrophilicity of cyclic aminal 6 with the Fukui function of the methylene bridges and (b) the HOMO-LUMO gap, which was calculated as the difference between the HOMO of the nucleophile (we used phenol 7i, which possessed a calculated HOMO energy of −0.34552 Hartree) and the LUMO of cyclic aminal 6 (Figure 4). In the first approach, the DFT B3LYP method at the 6-31G(d,p) level allowed us to obtain the Mulliken charges of the methylene bridges on the cyclic aminal. Because the differences between values were small, the electronic density and polarizability of the C-N bonds of the aminal moiety were similar in all of the methylene bridges for cyclic aminal 6 in the gas phase. However, Mulliken charges are not a good theoretical descriptor of electrophilicity. Thus, we used condensed Fukui functions, which are better theoretical descriptors. We applied the methodology proposed by Yang and Mortimer [33], which is based on a Mulliken population analysis and the following finite difference approximation:

(1) $$f_{1}=q\left(N\right)-q\left(N-1\right)$$

**Figure 4:**
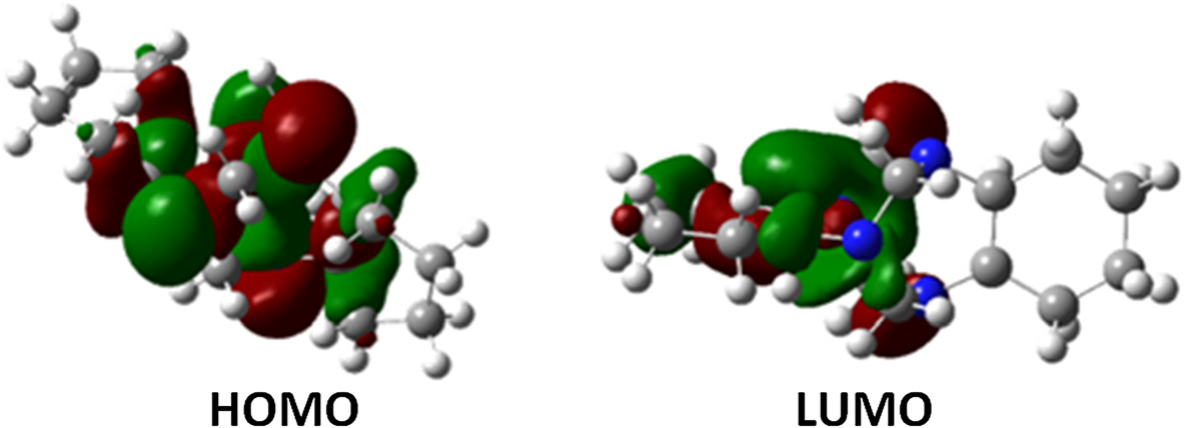
HOMO and LUMO tridimensional representation, calculated using Gaussian 1998 with DFT B3LYP at the 6-31G(d,p) level for cyclic aminal 6.

For a system of N electrons, independent calculations were made for the corresponding N and (N-1) systems with the same molecular geometry. According to this approach (Table 5), marked differences were observed in the electrophilicity of methylene bridges in aminal 6.

**Table 5:** Mulliken charges and Fukui function for 6 as electrophilicity theoretical descriptors

	**Mulliken charges**	**Fukui function**
C9	0.447	0.019
C18	0.447	0.019
C19	0.467	0.030
C20	0.356	0.027

Table 5 shows the results for computational calculations: Mulliken charges and Fukui function in each carbon atom of the cyclic aminal 6.

The condensed Fukui function for 6 suggests that the carbon atoms labeled as C19 and C20 are the most reactive sites for the nucleophilic attack of *p*-substituted phenol 7a-l (Figure 5). The calculated HOMO-LUMO gap between 6 and phenol 7i was 218.2 kCal/mol, which is consistent with the calculated Fukui indices, corroborating the electrophilic character of aminal 6. Furthermore, both the HOMO and LUMO in cyclic aminal 6 was influenced by aminal *cis* isomerism, such that the calculated HOMO representation for aminal 6 is indicative of a σ type interaction between the nonbonding molecular orbitals of the nitrogen atoms, which is favored by the eclipsed conformation as a result of *cis* isomerism (Figure 4).

**Figure 5:**
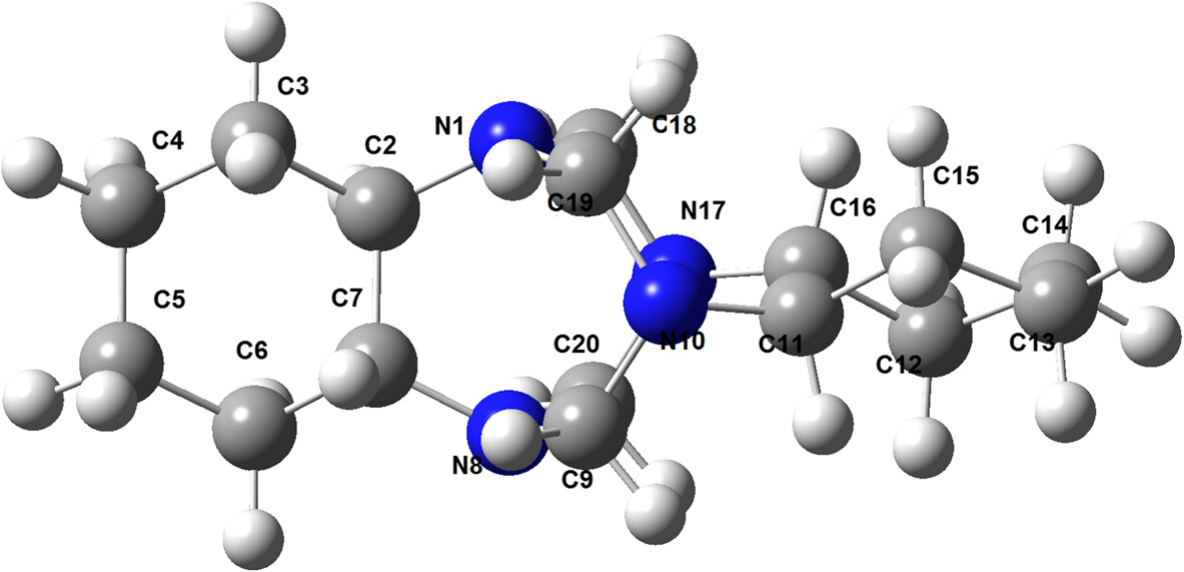
Atom labeling for compound 6.

Finally, we propose that the reaction between 6 and *p*-substituted phenols 7a-l is mediated by the hydrogen bond between any of the four nitrogen atoms in the cyclic aminal and the hydroxyl group of one molecule of phenol, in accordance with the mechanism for the reaction of cyclic aminal 1 previously reported in the literature [11].

### Conclusions

In summary, we synthesized a series of new *meso* Mannich bases called 4,4′-disubstituted-2,2′-{[(3a*R*,7a*S*)-2,3,3a,4,5,6,7,7a-octahydro-1*H*-1,3-benzimidazole-1,3-diyl]*bis*(methylene)} diphenols (8a-l) by reacting preformed Mannich reagent (6) with *p*-substituted phenols 7a-l. The presence of hydrogen bonding interactions in the molecular structure was demonstrated using X-ray diffraction, theoretical calculations and experimental spectroscopy. We demonstrated the synthetic potential of cyclic aminal 6 as a Mannich base precursor.

### Experimental

#### General and instrumentation

Melting points were determined with an Electrothermal apptus and are uncorrected. Diastereomerically pure *cis*-(*meso*)-1,2-diaminocyclohexane was purchased from Aldrich. (2*S*,7*R*,11*S*,16*R*)-1,8,10,17-tetraazapentacyclo[8.8.1.1.^8,17^ 0.^2,7^0^11,16^]icosane (6) was prepared according to the procedure described in the literature [27]. *p*-Substituted phenols (7a-l) were purchased from Merck and were used without further purification.

#### General procedure for the synthesis of compounds 8a-l

To a stirred solution of (2*S*,7*R*,11*S*,16*R*)-1,8,10,17-tetraazapentacyclo[8.8.1.1.^8,17^0.^2,7^0^11,16^] icosane (6) (276 mg, 1.00 mmol) in dioxane (3 mL), the respective *p*-substituted phenol (2.00 mmol) in dioxane (3 mL) was added dropwise. After stirring for 15 min at room temperature, water (4 mL) was added and the mixture was heated to 40°C for 30 h. After cooling to room temperature, the solvent was removed *in vacuo,* and the crude product was purified by chromatography on a silica column and subjected to gradient elution with light petroleum ether and ethyl acetate.

#### Single crystal X-ray measurements

Crystal data for compound 8j, C_23_H_30_N_2_O_2_, were collected using an Xcalibur Atlas Gemini ultra diffractometer using the following pmeters: Oxford Diffraction at 120 K, M_r_ = 366.5, triclinic, *P1*, a = 6.0346(4) Å, b = 12.4793(8) Å, c = 14.2169(10) Å, α = 67.847(7)°, β = 85.183(6)°, γ = 85.605(5)°, *V* = 986.93(12) Å^3^, *Z* = 2, Dx = 1.233 Mg m^-3^, CuKα X-ray source (radiation), λ = 1.5418 Å and F(000) = 396 colorless crystals 0.17 × 0.06 × 0.03 mm. All non-hydrogen atoms were refined with anisotropic thermal pmeters using full-matrix least squares procedures on *F*^*2*^ to give *R* = 0.036, *wR* = 0.086 for 2546 independently observed reflections and 251 pmeters. Crystallographic data (excluding structural factors) for the given structure in this article have been deposited at the Cambridge Crystallographic Data Centre (CCDC) as supplementary publication number CCDC 929464. Copies of these data can be obtained, free of charge, upon request to the CCDC at 12 Union Road, Cambridge. CB2 IEZ, UK. Fax: +44-(0)1223-336033 or e-mail: deposit@ccdc.cam.ac.uk. Program used to refine structure: JANA2006 [34].

### Competing interests

The authors declare that they have no competing interests.

### Authors’ contributions

AR conceived the study, participated in its design and coordination and helped draft the manuscript. DQ synthesized the compounds, performed the analysis, elucidated the structures and drafted the manuscript. JR-M participated in the development of theoretical models and computational analysis. EV collected the X-ray data and solved the crystal structure under the guidance of MD. All authors read and approved the final manuscript.

## References

[B1] Tramontini M Angiolini L Ghedini N Mannich bases in polymer chemistry Polymer 1988 29 5 771 788 10.1016/0032-3861(88)90132-2

[B2] Zhao Y-J Wei W Su Z-G Ma G-H Poly(ethylene glycol) prodrug for anthracyclines via *N*-Mannich base linker: Design, synthesis and biological evaluation Int J Pharm 2009 379 1 90 99 10.1016/j.ijpharm.2009.06.013 19540322

[B3] Gul M Atalay M Gul HI Nakao C Lappalainen J The effects of some Mannich bases on heat shock proteins HSC70 and GRP75, and thioredoxin and glutaredoxin levels in Jurkat cells Toxicol 2005 19 5 573 580 10.1016/j.tiv.2005.03.004 15896550

[B4] Karthikeyan MS Prasad DJ Poojary B Bhat KS Holla BS Kumari NS Synthesis and biological activity of Schiff and Mannich bases bearing 2,4-dichloro-5-fluorophenyl moiety Bioorgan 2006 14 22 7482 7489 10.1016/j.bmc.2006.07.015 16879972

[B5] Pajak J Rospenk M Maes G Sobczyk L Matrix-isolation FT-IR and DFT theoretical studies of the intramolecular hydrogen bonding in Mannich bases Chem 2006 320 2–3 229 238

[B6] Slowikowska J Beagley B Pritchard RG Wozniak K X-ray studies of the intra- and intermolecular H-bonding in two phenolic Mannich bases J. Mol. Struct 1994 317 1–2 99 110

[B7] Schilf W Szady-Chelmieniecka A Grech E Przybylski P Brzezinski B Spectroscopic studies of new Schiff and Schiff–Mannich bases of ortho-derivatives of 4-bromophenol J. Mol. Struct 2002 643 1–3 115 121

[B8] Brzezinski B Wojciechowski G Urjasz H Zundel G FT-IR study of the proton polarizability of hydrogen bonds and of the hydrogen-bonded systems in a di-Mannich base of 5,5′-dimethoxy-2,2′-biphenol J. Mol. Struct 1998 470 3 335 339 10.1016/S0022-2860(98)00384-6

[B9] Koll A Melikova SM Karpfen A Wolschann P Spectroscopic and structural consequences of intramolecular hydrogen bond formation in *ortho*-dimethylaminomethylphenol J. Mol. Struct 2001 559 1–3 127 145

[B10] Cummings TF Shelton JR Mannich reaction mechanisms J Org Chem 1960 25 3 419 423 10.1021/jo01073a029

[B11] Rivera A Gallo GI Gayón ME Joseph-Nathan P A novel Mannich type reaction using aminals in alkaline medium Synth 1993 23 20 2921 2929 10.1080/00397919308012614

[B12] Rivera A Ríos-Motta J Quevedo R Joseph-Nathan P Nuevos aspectos de la reacción tipo Mannich en medio básico de 1,3,6,8-tetrazatriciclo[4.4.1.1^3,8^]dodecano (TATD) con fenoles Rev 2005 34 2 105 115

[B13] Rivera A Nerio LS Ríos-Motta J Fejfarová K Dušek M 2,2′-[Imidazolidine-1,3-diyl*bis*(methylene)]diphenol Acta Cryst 2012 E68 o170 o171 10.1107/S1600536811053748 PMC3254510 22259455

[B14] Rivera A Nerio LS Ríos-Motta J Fejfarová K Dušek M 4,4′-Difluoro-2,2′-[imidazolidine-1,3-diyl*bis*(methy-lene)]diphenol Acta Cryst 2012 E68 o3043 o3044 10.1107/S1600536812040329 PMC3470394 23125807

[B15] Rivera A Nerio LS Ríos-Motta J Fejfarová K Dušek M 4,4′-Dimethyl-2,2′-[imidazolidine-1,3-diyl*bis*(methy-lene)]diphenol Acta Cryst 2012 E68 o3172 10.1107/S1600536812042808 PMC3515265 23284485

[B16] Duhamel L Patai S Supplement F: The Chemistry of amino, nitroso and nitro compounds and their derivatives The Chemistry of the Functional Groups. Part 2 1982 Chichester, UK: John Wiley & Sons 850 890

[B17] Rivera A Maldonado M Unexpected behavior of 6*H*,13*H*-5:12,7:14-dimethanedibenzo[*d*,*i*]-[1,3,6,8]- tetraazecine (DMDBTA) toward phenols DBTA Tetrahedron Lett 2006 47 7467 7471 10.1016/j.tetlet.2006.08.045

[B18] Jacobsen EN Asymmetric catalysis of epoxide ring-opening reactions Acc Chem Res 2000 33 6 421 431 10.1021/ar960061v 10891060

[B19] Trost BM Crawley ML Asymmetric transition-metal-catalyzed allylic alkylations: applications in total synthesis Chem Rev 2003 103 8 2921 2944 10.1021/cr020027w 12914486

[B20] Trindade AF Gois PMP Afonso CAM Recyclable stereoselective catalysts Chem Rev 2009 109 2 418 514 10.1021/cr800200t 19209946

[B21] Yu CW Kay KW Li KK Pang SK Steve CF Au-Yeung SC Ho YP Anticancer activity of a series of platinum complexes integrating demethylcantharidin with isomers of 1,2-diaminocyclohexane Bioorgan 2006 16 6 1686 1691 10.1016/j.bmcl.2005.12.019 16386904

[B22] Xu Q Khokhar AR Synthesis and characterization of *N*-methyliminodiacetato *trans*-*R*,*R*-, *trans*-*S*,*S*-, and *cis*-1,2-diaminocyclohexane platinum (IV) complexes: Crystal structure of chloro(*trans*-*R*,*R*-1,2-diaminocyclohexane) (*N*-methyliminodiacetato)platinum(IV) chloride J Inorg Biochem 1992 48 3 217 226 10.1016/0162-0134(92)84032-I 1447569

[B23] Fernández-Fernández MC Bastida de la Calle R Macías A Valencia L Pérez-Lourido P Co(II), Ni(II) and Cu(II) complexes of new [1+1] and [2+2] macrocyclic ligands derived from 1,4-*bis*(2′-formylphenyl)-1,4-dioxabutane and *cis*-1,2-diaminocyclohexane Polyhedron 2008 27 11 2301 2308 10.1016/j.poly.2008.04.043

[B24] Yoshikawa K Yokomizo A Naito H Haginoya N Kobayashi S Yoshino T Nagata T Mochizuki A Osanai K Watanabe K Kanno H Ohta T Design, synthesis, and SAR of *cis*-1,2-diaminocyclohexane derivatives as potent factor Xa inhibitors. Part I: Exploration of 5–6 fused rings as alternative S1 moieties Bioorgan Med 2009 17 24 8206 8220 10.1016/j.bmc.2009.10.023 19884015

[B25] Yoshikawa K Kobayashi S Nakamoto Y Haginoya N Komoriya S Yoshino T Nagata T Mochizuki A Watanabe K Suzuki M Kanno H Ohta T Design, synthesis, and SAR of *cis*-1,2-diaminocyclohexane derivatives as potent factor Xa inhibitors. Part II: Exploration of 6–6 fused rings as alternative S1 moieties Bioorgan Med 2009 17 24 8221 8233 10.1016/j.bmc.2009.10.024 19900814

[B26] Murray-Rust P Riddell FG The stability and conformation of the 1,3,6,8-tetraazatricyclo[4.4.1.1^3,8^]dodecane system: the structure of the condensation product of 1,2-diaminocyclohexane and formaldehyde Can J Chem 1975 53 1933 1935 10.1139/v75-269

[B27] Glister JF Vaughan K Biradha K Zaworotko MJ (2*S*,7*R*,11*S*,16*R*)-1,8,10,17-tetraazapentacyclo [8.8.1.1^8,17^.0^2,7^.0^11,16^]icosane and its enantiomer. Synthesis, NMR analysis and X-ray crystal structure J 2005 749 78 83 10.1016/j.molstruc.2005.03.043

[B28] Frisch MJ Trucks GW Schlegel HB Scuseria GE Robb MA Cheeseman JR Zakrzewski VG Montgomery JA Jr Stratmann RE Burant JC Dapprich S Millam JM Daniels AD Kudin KN Strain MC Farkas O Tomasi J Barone V Cossi M Cammi R Mennucci B Pomelli C Adamo C Clifford S Ochterski J Petersson GA Ayala PY Cui Q Morokuma K Malick DK GAUSSIAN 98 (Revision A.9) 1998 Pittsburgh: PA: Gaussian Inc

[B29] Majerz I Dziembowska T Ambroziak K Analysis of the vibrational spectra of *trans-N,N´*-*bis-R*-salicylidene-1´,2′-cyclohexanediamine J 2007 828 166 173 10.1016/j.molstruc.2006.05.051

[B30] Zierkiewicz W Michalska D Molecular structure and infrared spectra of 4-fluorophenol: A combined theoretical and spectroscopic study J Phys Chem A 2003 107 4547 4554 10.1021/jp022564q

[B31] Navarro-Vazquez A Cobas JC Sardina FJ Casanueva J Díez E A Graphical tool for the prediction of vicinal proton-proton ^3^*J*_HH_ coupling constants J 2004 44 5 1680 1685 10.1021/ci049913t 15446826

[B32] Karplus M Vicinal proton coupling in nuclear magnetic resonance J Am Chem Soc 1963 85 18 2870 2871 10.1021/ja00901a059

[B33] Yang W Mortimer WJ The use of global and local molecular pmeters for the analysis of the gas-phase basicity of amines J Am Chem Soc 1986 108 19 5708 5711 10.1021/ja00279a008 22175316

[B34] Petřίček V Dusěk M Palatinus L JANA2006 2006 Praha, Czech Republic: Institute of Physics

